# Near Real-Time Syndromic Surveillance of Emergency Department Triage Texts Using Natural Language Processing: Case Study in Febrile Convulsion Detection

**DOI:** 10.2196/54449

**Published:** 2024-08-30

**Authors:** Sedigh Khademi, Christopher Palmer, Muhammad Javed, Gerardo Luis Dimaguila, Hazel Clothier, Jim Buttery, Jim Black

**Affiliations:** 1 Department of Paediatrics University of Melbourne Melbourne Australia; 2 Health Informatics Group Centre for Health Analytics Melbourne Children's Campus Melbourne Australia; 3 SAEFVIC Murdoch Children’s Research Institute Melbourne Australia; 4 Melbourne School of Population and Global Health University of Melbourne Melbourne Australia; 5 Infectious Diseases Royal Children’s Hospital Melbourne Australia; 6 Department of Health State Government of Victoria Melbourne Australia

**Keywords:** vaccine safety, immunization, febrile convulsion, syndromic surveillance, emergency department, natural language processing

## Abstract

**Background:**

Collecting information on adverse events following immunization from as many sources as possible is critical for promptly identifying potential safety concerns and taking appropriate actions. Febrile convulsions are recognized as an important potential reaction to vaccination in children aged <6 years.

**Objective:**

The primary aim of this study was to evaluate the performance of natural language processing techniques and machine learning (ML) models for the rapid detection of febrile convulsion presentations in emergency departments (EDs), especially with respect to the minimum training data requirements to obtain optimum model performance. In addition, we examined the deployment requirements for a ML model to perform real-time monitoring of ED triage notes.

**Methods:**

We developed a pattern matching approach as a baseline and evaluated ML models for the classification of febrile convulsions in ED triage notes to determine both their training requirements and their effectiveness in detecting febrile convulsions. We measured their performance during training and then compared the deployed models’ result on new incoming ED data.

**Results:**

Although the best standard neural networks had acceptable performance and were low-resource models, transformer-based models outperformed them substantially, justifying their ongoing deployment.

**Conclusions:**

Using natural language processing, particularly with the use of large language models, offers significant advantages in syndromic surveillance. Large language models make highly effective classifiers, and their text generation capacity can be used to enhance the quality and diversity of training data.

## Introduction

### Background

A febrile convulsion refers to a seizure triggered by a fever, most commonly experienced by children aged between 6 months and 5 years, in the absence of an underlying central nervous system infection or metabolic disturbance [1]. Febrile convulsions have various causes and risk factors, including viral or bacterial infections, a family history of seizures, underlying neurological conditions, environmental factors, and specific vaccinations [2]. Age, fever, and a seizure are essential components of the definition of childhood febrile convulsion [3]. Febrile convulsions are most often caused by viral respiratory tract infections but are also associated with viral infections such as chicken pox, tonsillitis, and middle ear infections. Febrile convulsions are also associated with the administration of childhood vaccines [4]. Although febrile convulsions caused by vaccines are rare and typically do not cause permanent damage, parents’ experiences with their children’s febrile convulsions can have a negative effect on their perception of vaccine safety [5].

In 2010, in Australia, there was an increase of febrile convulsions in young children after the release of the southern hemisphere trivalent inactivated influenza vaccine, produced by CSL Biotherapies [6,7]. Following national suspension of seasonal influenza vaccinations for children aged <5 years, reviews [8,9] revealed deficiencies in Australian adverse event following immunization (AEFI) monitoring system, which had resulted in delayed reporting and underreporting of febrile convulsions [10,11]. The reviews highlighted the need for monitoring additional data sources for early AEFI detection, a subsequent focus of Surveillance of Adverse Events Following Vaccination In the Community [12,13] and the Health Informatics groups at the Murdoch Children’s Research Institute, Victoria, Australia. A recent paper highlighted the need for vaccine safety monitoring to include natural language processing (NLP) of both internet-based data sources and electronic health records [14].

In this study, we aimed to assess the effectiveness of NLP techniques for rapid detection of febrile convulsion presentations in emergency departments (EDs).

Syndromic surveillance relies on the categorization of patient-presented symptoms and complaints into “syndromic indicators,” often derived from patient-reported or observed symptoms [15]. These indicators, recorded by health care providers during the initial patient contact, along with preliminary or working diagnoses, are crucial in the absence of any confirmatory testing or diagnosis to facilitate prompt public health decisions [16]. Examples include monitoring of telephone health advice systems, of notes taken during attendance to primary physicians, and of data entry performed during visits to ED.

Syndromic surveillance has shown to have the ability to rapidly evaluate the potential impact of a recently introduced vaccine [17,18]. Monitoring telephone helpline data can also assist with early detection of AEFI, and in the case of 2010 Australian AEFI signal, retrospective analysis of these data showed that such methods would have flagged a signal 2 weeks after commencement of vaccination, which is 4 weeks earlier than the alert was raised [19]. Surveillance of ED triage notes is particularly effective for timely syndromic information capture, as data are entered upon a patient’s arrival to ED, allowing for the initiation of a notification from a surveillance system while the patient is still in the ED [20], well before any diagnostic coding takes place.

ED triage notes are gathered during the first moments of the patient encounter and usually contain aspects of a patient’s medical history, presenting symptoms, and the reasons for their visit. This information is primarily used to direct initial clinical management and can serve as a tool to help understand trends of patient visits in near real time [21]. However, variation in the language used in the documentation of this information within and across hospitals significantly impedes the reuse of these data [22]. Abbreviations abound and their meanings vary according to context; for example, “cp” might be used as abbreviation for any type of *chest* pain, which can include pulmonary and trauma-related sources, in some contexts, it might just mean *cardiac* pain, while in others, it may refer to cerebral palsy. In some presentations, “NVD” means “nausea, vomiting, and diarrhea,” but in relation to childbirth, “NVD” means “normal vaginal delivery.” Misspellings, local variations of abbreviations, and context-sensitive vocabulary all feature in ED notes, and there are additional variations of the quality and length of the texts [23].

Research examining triage notes can be broadly classified into 3 main categories: quality improvement of triage notes’ recording and category assignment, prediction, and case identification. Studies focusing on the quality improvement of triage notes’ recording and category assignment aim to enhance the accuracy, reliability, efficiency, and completeness of the information recorded during triage. Prediction studies aim to predict the outcomes of emergency visits or the resources needed by patients based on the information recorded in triage notes. Case identification studies aim to either classify ED visits into categories or syndromes or to collect data about specific presentations (or syndromes) of interest [24].

Various methods have been used for identifying syndromes from triage notes. These include keyword-based, linguistic-based, statistical and machine learning (ML) algorithms, or hybrids of these [25]. Some have used data from 1 hospital [26], while others have used data from >1 hospital [27]. These systems vary in their goals, such as focusing on classification of 1 syndrome [28] or developing a syndromic surveillance system for >1 syndrome [29].

In recent years, the use of ML algorithms for surveillance of ED triage notes has increased [24]. One of the main obstacles in using supervised ML algorithms is the scarcity of annotated data for training and benchmarking [30]. Many studies have used medical coding against existing data as a proxy for the labels [31-34]. However, the use of *International Classification of Diseases* codes as a gold standard has known limitations as they do not always align with the actual reason for the visit [35]. For instance, codes can be assigned to identify the underlying etiology of a presentation, for health conditions not directly observed in the text of a presentation, or for other purposes such as financial incentives [36,37]. The choice of codes can be influenced by perceptions of the importance of a certain condition [38], and in this study, a febrile convulsion might not get coded if it is thought of as a secondary effect or not significant enough to include on a discharge summary when there are limitations to how many codes may be assigned.

When using supervised ML algorithms in the context of syndromic surveillance of ED triage notes, researchers have manually annotated from several thousand [29] to a few hundred thousand records [39] to train the algorithms. It has been observed that data annotation poses a significant obstacle in training NLP models within the clinical domain, with manual identification of labels affecting the representativeness of samples. This challenge often restricts NLP solutions to obtain data from only a few institutions, thereby impacting their generalizability [40].

### Objectives

In this study, the overarching aim was to identify emerging trends that could signal potential issues with a vaccine. Our primary objective was to construct a highly effective NLP model for the early detection of febrile convulsions in ED notes, applicable to the entirety of public hospital ED departments, without requiring large volumes of annotated training data. We achieved this goal by leveraging a limited set of manually labeled records and using data augmentation techniques. Our data set was sourced from 26 public hospitals across the Australian State of Victoria. Furthermore, we aimed to outline the essential requirements for the development and deployment of such a system.

## Methods

### Data

#### Overview

SynSurv provided the primary data source for this study. SynSurv is the syndromic surveillance project of the Department of Health of the state government of Victoria, Australia. Its objective is to detect events of public health significance early, allowing clients responsible for public health action to respond promptly and effectively. At the time of writing, SynSurv receives a rapid stream of information about every ED presentation, including the triage text, from a majority (n=34) of the public hospitals with Emergency Departments in Victoria, Australia. Most presentations arrive within 5 to 15 minutes of the patient’s assessment.

Data comprise the text recorded at triage by ED nurses and are characterized by a unique structure that primarily consist of abbreviations and brief phrases. The text usually contains a presenting complaint, selected past medical history, and the nurse’s observations of the patient. Triage text does not contain demographic or identifying information. The length of the text varies; it may be a detailed narrative of the patient’s presentation to the triage nurse or it could be a concise summary of a possible diagnosis along with a few observations. The unlabeled data set used in this study consisted of 76,274 ED triage text from January 1 to July 14, 2022, of ED presentations of children aged between 6 months and 6 years. The average length of text was 22 (SD 20.4) words. The longest record initially contained 319 words, but after data preparation, which involved removing nontextual information, the length of the longest text was reduced to 253 words. Additional data collected in 2022 were used to create a hold-out test data set.

#### Febrile Convulsion Symptoms

During the initial, “tonic” seizure stage of a febrile convulsion, the individual may let out a cry or moan before suddenly losing consciousness and experiencing muscular rigidity. This stage can last for up to 30 seconds and may be accompanied by the cessation of respiratory movements. The “clonic” seizure stage that follows involves repetitive movements of the limbs or face. While rigors (uncontrolled shivering and shaking) may look similar and often occur during any acute febrile illness, loss of consciousness is not typically associated with them [41]. Seizures typically last <5 minutes, although they may be prolonged. A “postictal state” follows, lasting between 5 and 30 minutes, during which the patient can experience drowsiness, confusion, headaches, and nausea while gradually returning to normal.

#### Data Annotation

Annotation of febrile convulsions needs to account for the language used in their clinical descriptions, which includes temperature-related terms and terms used to describe the clonic, tonic, and postictal stages of a seizure.

The first step of annotation involved filtering the ED notes for convulsion-related terms (eg, “seiz,” “convuls,” “fit,” “epilep,” “ictal,” “tonic,” or “clonic”) and fever-related terms (eg, “febri,” “fever,” “37.”, “38.”, “39.”, “40.”, “41.”, “42.”, or “43.”). Applying the filter reduced the data set to around 29,000 candidates for labeling, and these were annotated with a goal to identify around 1000 positive examples of febrile convulsion. The ED nurse’s notes were thoroughly reviewed, and if there was a likely indication of a febrile convulsion, whether explicitly mentioned or not, a positive label was assigned by J Black (described below), and a negative label was assigned to records that did not meet the criteria. In an additional step, some records that did not contain any of the filter settings were randomly selected and labeled. Only a few of these were identified as positive, mostly due to spelling variations in the filter strings that caused the records not to be detected in the initial step.

An annotation guideline was developed by J Black, who is a physician with ED experience, where a record was labeled as positive if the following criteria were met:

- The patient presented with febrile convulsion symptoms at the time of ED presentation, which requires mentions of both seizure and fever.
- The mention of febrile convulsion is not just in the patient’s medical history (eg, only “phx febrile convulsion”) or just an expression of parental concern (eg, only “mother worried as child previously had a seizure”).
- The convulsion is not related to other chronic conditions that include seizures, as febrile illness can trigger a pre-existing disposition to seizures. A mention of medicine usually taken when seizures happen is an indication of existing underlying cause.
- A mention of fever-lowering medications, subjective assessment of fever by parents or carers, or measurements taken at home can indicate the presence of a fever, even if the temperature recorded in the ED is normal.
- The notes do not indicate other types of seizure, including absent or focal seizures.

Following this guideline, author J Black annotated the training data to classify instances as either febrile convulsion or not. This resulted in 1032 positive labels and 14,415 negative labels, making a total of 15,447 annotations. The annotation of the separate test data set resulted in 432 positive and 2768 negative labels, a total of 3200 records. Table 1 provides examples of triage notes along with their corresponding labels, and Table 2 enumerates the record and word counts of the data sets.

**Table 1:** Sample of triage notes (not the actual text but examples of typical structure).

Label	Category	Triage notes
1	Fever and seizure present	“Seizure, Unwell Since yesterday, Febrile, Vomit enroute, IUTD, Nil Rash, A-PATENT, Nil Sob, T- 38.8, GCS-15, R- 22, Nil Pain, PWD, O/A alert.”
1	“Tonic clonic” and fever but no mention of convulsion or seizure	“BIBA: Unwell 1/12 (fev,diahhroea). 1× ep of eye rolling back? tonic clon. Self res 1/60. Good oral intake. O/E: PWD, Good capp Asleep, easily rouse. Ket 0.4 Pmhx: UTDI”
1	Fever and spelling variation of seizure-related term and fever	“FEVER. Decreased oral intake. Sz today. Runny nose. Clear chest. Nil vomiting. Miserable at triage. Phx dad states rare gene mutation.”
1	Seizure and spelling variation of fever-related term	“BIBA post seizure tonight, eyes rolled and floppy unresponsive 5 min. Temps last 3/52. At triage febrile.”
1	Seizure and fever medication is mentioned	“At midnight Advil given. At 0100 10 sec of seizure like activity. 2nd seizure activity shortly after lasting a few minutes with bilious vomit. O/A alert. RR 24 sats 99 no WOB HR 112.”
0	Underlying condition for seizure	“Prolonged sz at home lasting 13 mins. Congested left lung with resp symptoms. Runny nose and cough this week. 4mg oral midaz. Resolved with av arrival. Postictal 40 mins. Phx sz focal or tonic/clonic phx eplispey and dravat sx”
0	Febrile convulsion in past medical history only	“BIBA seizure like activity tonight, intermittent 15 mins, post ictal .30/60. Hx febrile convulsions. Afebrile oa”
0	Parental concerns	“FEVER Since yesterday, Coughing, Nasal congestion, Parents concerned as previous febrile convulsion”
0	Other types of seizures	“FEVER? Absent episode at day care following fever. Nil seizure activity, but more quiet. GCS 15. pHx asthma”

**Table 2:** Descriptive statistics of the data set.

Data set	Total records, n	Total words, n	Average words, n	Unique words, n
Initial data	76,274	1,654,045	21.69	52,698
Training data	15,447	398,608	25.80	21,086
Test data	3200	102,625	32.07	9925

#### Data Set Construction

The original data were very imbalanced, with 1032 positive labels versus 14,415 negative labels. Our approach was to allow for the influence of the negative examples as much as the positive examples, as we wanted to ensure the models were not overly prone to identify false positives. We decided to evaluate as many negatively labeled records as possible by dividing the negative data into smaller data sets that each roughly matched the number of available positive records, while also matching the positive records’ text lengths, and we paired each of these with the positive records for a training data set. To accomplish text length matching, we assigned a word-count group to each record, and when sampling negative records, we ensured that we took similar numbers from each word-count group to those which existed in the positive records.

After setting aside a validation data set of 100 positive and 100 negative labels, 932 positive labels remained for training. When sampling the negative labels to match the 932 positive labels for a training data set, we oversampled by a factor of around 1.2, iteratively extracting negative examples until we ran out of examples to complete a training data set. The result was 9 data sets each consisting of the same 932 positive labels and 1127 different negative labels, with a total of 2059 records in each. These were used for initial training of 9 identical transformer models, and by assessing their test scores, we could determine that the best model also identified the best of the 9 training data sets, where the balance of negative examples worked most advantageously with the positive examples. We chose transformers for the training data evaluation because with their capacity to take account of language structure, they were more sensitive to textual information compared to the other standard neural networks we were evaluating [42].

#### Data Augmentation

When evaluating the transformer models, we found that there was potential for improvement in their performance, as their *F*_1_-score was around 0.79. Therefore, we decided to assess the effect of training with additional examples. As we lacked new positive examples, we decided to experiment with data augmentation techniques. These included synthetic text generation using GPT-2 models, domain-specific data augmentation, and task-agnostic techniques. This is explained in a prior publication [43] where the best result was achieved by using synthetic text generation techniques. Using this approach added 1582 positive labeled records to the training data set. This meant we could safely add further negative examples without the data set becoming imbalanced, resulting in 5455 training records. We used this augmented data set and the 200-record validation data set to train and validate all the approaches, and we evaluated and compared them using the 3200-record holdout test set.

After conducting error analysis on the predictions of the transformer model, we added another 112 seizure-related negative labeled records to the data set. This gave the models additional exposure to ED notes about other types of seizures or tonic-clonic seizures without the fever components, which would allow to the model to better learn not to create false positive predictions on these marginally negative examples. The final training data set consisted of 2514 positive and 3053 negative labels, a total of 5567 records. Table 3 shows the construction of the data sets.

**Table 3:** Data sets’ construction.

Data set	Positive, n (%)	Negative, n (%)
Initial training (×9; n=2059)	932 (45.3)	1127 (54.7)
Augmented training (n=5455)	2514 (46.11)	2941 (53.89)
Final training (n=5567)	2514 (45.22)	3053 (54.78)
Validation (n=200)	100 (50)	100 (50)
Test (n=3200)	432 (13.5)	2768 (86.5)

### Classification

#### Overview

As part of our goal of determining the most effective NLP methods for identifying febrile convulsions in ED visits, we needed to assess the trade-off in requirements and benefits of increasingly sophisticated NLP models. While the Data section described the data requirements that evolved as these models were assessed, the Classification section describes the reasons for evaluating the various models, their data preparation and training requirements, and some relevant observations of their training processes. Evaluation and results will be discussed fully in the Results section.

#### Pattern Matching

We started with pattern matching as a manual approach that could give us a baseline against which we could compare ML approaches. This consisted of selecting text based on relevant strings that would, when combined, indicate both fever and convulsions. After importing the data into a Structured Query Language (SQL) database, we used a SQL full-text search to describe the patterns more comprehensively and to ensure that both fever and convulsions were included together. The pattern used was (‘ “fever*” OR “pyrexi*” OR “feb*” OR “37.6” OR “37.7” OR “37.8” OR “37.9” OR “38.*” OR “39.*” OR “40.*” OR “41.*” OR “42.*” OR “43.*” OR “T38*” OR “Temp38*” OR “T39*” OR “Temp39*” OR “T40*” OR “Temp40*” OR “T41*” OR “Temp41*” OR “T42*” OR “Temp42*” OR “T43*” OR “Temp43*” OR “hot*” OR “warm*” ’) AND (‘ “convul*” OR “*seiz*” OR “size*” OR “*sezi*” OR ictal OR tonic OR clonic ’). We experimented with accounting for negations and modifiers, such as mentions of medical history and temperature measurement units, when they were close to terms related to febrile convulsions. Although this improved the detection of false positives, it detrimentally affected the detection of true positives and ultimately resulted in a poorer performance of the model. Therefore, to retain the simplest and most performant pattern matching approach, we decided to avoid dealing with negations and modifiers.

#### Standard Classifiers

We evaluated a variety of standard classifiers using both the original text with trigrams and lemmatized forms of trigrams. Given the highly specific nature of the texts, where abbreviations and punctuations prevail, we did not eliminate stop words, and we restricted our preprocessing to (1) expanding any contractions that used apostrophes and (2) converting collections of the plus sign (eg, “+++”) into the word “extreme,” as these are used throughout to convey that meaning. We used a customized tokenization method, based on spaCy, to ensure that tokenization did not break apart symbols on embedded periods and slashes and to fashion bigrams, trigrams, and lemmatized words. This was because we needed to preserve the forms of words, especially regarding temperatures and explicit compound expressions, and to control how n-grams and lemmatization were performed. We experimented with the Scikit-Learn CountVectorizer and TfidfVectorizer, with and without inverse document frequency (IDF) enabled; with the result that for each of the 3 data sets, we had standard, trigram, and lemmatized versions, each of which were assessed via a CountVectorizer, TfidfVectorizer with IDF, and TfidfVectorizer without IDF enabled; a total of 27 data sets for each of the classifiers were assessed.

#### Standard Neural Networks

We preprocessed the data for experimentation with standard neural networks by separating basic contractions (eg, isn’t to is n’t), but otherwise, the data were left intact, with no ED-specific translations done per the Standard Classifiers section, such as “+++” to “extreme.” The preprocessed text was tokenized using the *torchtext* library, and models were constructed using the Pytorch library. We experimented with a range of neural network models—convolutional neural network (CNN), long short-term memory (LSTM), bidirectional LSTM (BiLSTM), CNN-LSTM, and CNN-BiLTSM hybrids. Then, we tested the gated recurrent unit (GRU), bidirectional GRU (BiGRU), and CNN-GRU and CNN-biGRU hybrids. We implemented the best of these—the BiGRU and the CNN-BiGRU hybrid. The latter consisted of a 3-layer CNN producing one-grams, bigrams, and trigrams (via kernel sizes of 1, 2, and 3 with a kernel number of 100), with its output concatenated with a BiGRU output. The standard BiGRU model’s *F*_1_-score slightly exceeded that of the CNN-BiGRU hybrid model, but the CNN-BiGRU was included because it mostly had a better balance of precision and recall and was a strong contender.

#### Transformers

We used the RoBERTa-large-PM-M3-Voc model, published by Facebook [44] and described as being “pre-trained on PubMed and PMC and MIMIC-III with a BPE Vocab learnt from PubMed.” This model was selected due to its superior performance in classifying biomedical and clinical texts compared to other models with similar capabilities, including Scientific Bidirectional Encoder Representations from Transformers (SciBERT) by the Allen Institute for Artificial Intelligence, Biomedical (BioBERT) by researchers at Korea University and the National Institutes of Health, ClinicalBERT by researchers at the University of Pennsylvania and the University of Washington, and BioMed-RoBERTa by researchers at the University of California, San Diego. We did no text preprocessing, as we considered that the transformer’s internal byte pair encoding approach and inherent language understanding as sufficient to deal with the texts’ complexity. The best transformer model was identified from the final form of the training data.

### Ethical Considerations

Ethics approval for this study was granted by the Department of Health, Human Research Ethics Committee in Victoria, Australia (project ID: HREC/83486/DOH-2022-298485). No compensation was provided to any participants. Informed consent was not sought for this study because the operational work it supports aligns with legislation related to serious public health threats. The data were anonymized by removing personal details and using a 1-way hashing algorithm to ensure that reidentification is not possible.

## Results

### Overview

The results are shown in Table 4 as precision, recall, and *F*_1_-scores when evaluated against the test data set. We used precision, recall, and *F*_1_-scores as evaluation metrics to assess the performance of the models on the positive label using the test data set. Precision measures the proportion of correctly classified positive instances out of all instances predicted as positive. Recall measures the proportion of correctly classified positive instances out of all actual positive instances. *F*_1_-score is the harmonic mean of precision and recall, providing a balanced measure of performance. By using these evaluation metrics, we were able to comprehensively evaluate the models’ performance on key measures of accuracy and completeness with respect to both the positive and negative labels.

Scores are depicted for each model in order of the data sets used to train a model. These were (1) the best of the initial training data set of 2059 records, (2) the synthetic records–enhanced data set of 5455 records, and (3) the data set also containing 112 additional examples of negative seizure examples, with a total of 5567 records. These data sets are indicated with superscripts of b, d, and e in Table 4.

The models’ difference scores are shown at the bottom of each model group. These are calculated as the difference between model test scores obtained when trained on the final (ie, third) data set (superscript e) and the test scores obtained using the pattern matching approach, which functions as a baseline. The best individual value in each of the table columns are in italics, but the *F*_1_-score is the most important value to measure overall performance.

**Figure 1:**
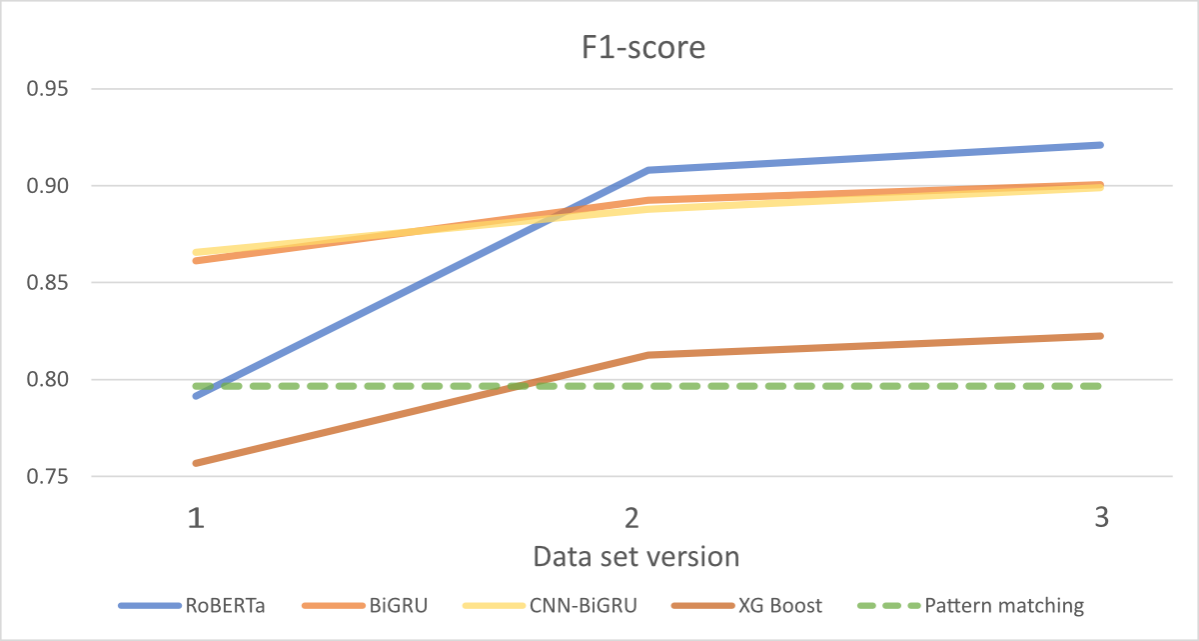
Comparison of the *F*_1_-scores of the models. BiGRU: bidirectional gated recurrent unit; CNN-BiGRU: convolutional neural network-bidirectional gated recurrent unit; RoBERTa: Robustly optimized Bidirectional Encoder Representations from Transformers approach; XGBoost: extreme gradient boosting.

Figure 1 shows a graphical comparison of the *F*_1_-scores achieved per model on the test data set, as the models were trained on the 3 training data sets. Notably, the *F*_1_-score of the RoBERTa transformer model was initially no better than pattern matching when trained on the first data set. However, as more data were added, the RoBERTa transformer model’s performance improved significantly, surpassing the *F*_1_-scores of the other models starting from the second data set onward.

**Table 4:** Model performance metrics.

Model and data sets	Precision	Recall	*F*_1_-score	True negative	True positive	False positive	False negative
Pattern matching	0.665	*0.993^a^*	0.797	2552	*429*	216	*3*
RoBERTa^b,c^	0.658	*0.993*	0.792	2545	*429*	223	*3*
RoBERTa^d^	0.852	0.972	0.908	2695	420	73	12
RoBERTa^e^	0.875	0.972	*0.921*	2708	420	60	12
RoBERTa—difference^e^	0.210	–0.021	0.124	156	–9	–156	9
BiGRU^b,f^	0.812	0.917	0.861	2675	397	92	36
BiGRU^d^	0.866	0.921	0.893	2705	399	62	34
BiGRU^e^	*0.903*	0.898	0.900	2725	389	*42*	44
BiGRU—difference^e^	0.237	–0.095	0.104	173	–40	–174	41
CNN-BiGRU^b,g^	0.822	0.915	0.866	2681	396	86	37
CNN-BiGRU^d^	0.889	0.887	0.888	2719	384	48	49
CNN-BiGRU^e^	0.864	0.937	0.899	*2742*	374	59	25
CNN-BiGRU—difference^e^	0.199	–0.056	0.102	190	–55	–157	22
XGBoost^b,h^	0.633	0.940	0.757	2533	406	235	26
XGBoost^d^	0.723	0.928	0.813	2614	401	154	31
XGBoost^e^	0.746	0.917	0.822	2633	396	135	36
XGBoost—difference^e^	0.081	–0.076	0.026	81	–33	–81	33

^a^Italicized values represent the best individual value in each of the columns.

^b^The best of the initial training data set of 2059 records.

^c^RoBERTa: Robustly optimized Bidirectional Encoder Representations from Transformers approach.

^d^The synthetic records–enhanced data set of 5455 records.

^e^The data set also containing 112 additional examples of negative seizure examples.

^f^BiGRU: bidirectional gated recurrent unit.

^g^CNN-BiGRU: convolutional neural network-bidirectional gated recurrent unit.

^h^XGBoost: extreme gradient boosting.

### Pattern Matching

The pattern matching method we assessed was a rules-based approach looking at text patterns in a SQL full-text query. It achieved a very high recall of 0.99—meaning it correctly identified almost all the febrile convulsion records. However, it also identified many incorrect records as febrile convulsions with a resulting precision of 0.67, so its *F*_1_-score suffered, although it remained acceptable at 0.80 (rounded). Analysis of false positives showed that mentions of time of the day, duration of seizure, and child weight were all open to misinterpretation as indications of fever, as numbers ranging from “37.6” through to “43.” were matched as indicators of temperature. By contrast, references to temperature that had no accompanying qualifier or decimal point were ignored. Terms such as “warm to touch” were missed, where warm can refer to fever (but is also used in observations about skin being pink, warm, and dry). Imprecise descriptions of febrile convulsion, negations, and mentions of previous history of fever, seizure, and febrile convulsions were missed or misinterpreted. For instance, implied and specific references to history such as “increased seizure,” “mother concerned,” “Mum states older brother has had a febrile seizure before,” “febrile convulsion previously,” “recent admission with seizure + enterovirus,” and mentions of use of seizure-related medications before the emergency visit. We experimented with including pattern matching of previous history in our query, which worked to a certain extent to remove false positives but also removed true positives, with a resulting worse performance. A fully implemented pattern matching system requires extensive rules that adjust for many textual nuances but which can never be complete and would be difficult to maintain, which is why we focused our effort on ML solutions.

### Standard Classifiers

We assessed the Scikit-Learn Multinomial Naive Bayes, logistic regression cross validation, linear support vector classification (SVC), stochastic gradient descent (SGD), random forest, extra trees classifiers, and the extreme gradient boosting (XGBoost) classifier. Each was tested with the 3 data sets; with the standard form of the text, with trigrams, and with lemmatized trigrams; and vectorizing with the Scikit-Learn CountVectorizer, TfidfVectorizer with IDF, and TfidfVectorizer without IDF enabled. Grid searches were performed to further tune model parameters for the best models from each round. We evaluated the models with the test data set.

On the initial data set of 2059 records, the best model was the XG Boost classifier, using lemmatized text and the CountVectorizer, with an *F*_1_-score of 0.757. The second best was the logistic regression cross validation model, using standard text and the CountVectorizer, with an *F*_1_-score of 0.755. The XG Boost model continued to be the best model as we assessed with the larger data sets. With the augmented, second data set of 5455 records, which included synthetic positive examples, the *F*_1_-score was 0.813, using standard text and the TfidfVectorizer with no IDF. With the third data set of 5567 records, which included extra negative seizure examples, the *F*_1_-score was 0.822, again using standard text and the TfidfVectorizer but this time with IDF enabled.

The standard classifiers were worse than pattern matching on the initial data set, but on the larger data sets, the best of the models scored better than pattern matching. The XG Boost model fared well for *recall*, with an average of 0.93 over the data sets, which was marginally better than the standard neural networks’ average of 0.91 but not as good as the transformer’s average of 0.98. However, the superior precision of the standard neural networks resulted in their *F*_1_-scores being, on average, 0.09 higher than those of the XGBoost model, while the average *F*_1_-score of the transformer model was 0.10 higher. With a relatively good recall but overall poorer performance and a high degree of effort required to prepare for and assess these models, we would consider these only in a low-resource situation and would consider pattern matching as a comparable option.

### BiGRU and CNN-BiGRU

The BiGRU and CNN-BiGRU classifiers were Pytorch models trained from scratch on the different data sets. Other models that were evaluated did not come close to their performance; these included a CNN model, LSTM models (in both standard and bidirectional form), CNN-LSTM, CNN-BiLSTM, and a BiGRU model with an additional attention layer. Word2Vec embeddings from the training data sets were loaded into the models.

Their performance based on *F*_1_-score was as much as 10% better than that of pattern matching at 0.8, ranging from around 0.86 to around 0.9 as the data sets were developed. This was because their precision was much better; they found fewer false positives, although their recall was poorer compared to pattern matching by as much as 10%.

Initially, they were better than the transformer approach as well; their starting *F*_1_-score of around 0.86 exceeded the transformer’s score of 0.79. However, as data were added, first through additional synthetic positive records and then by adding negative seizure examples, these models only improved over their initial scores by around 4 percentage points (lower than the improvement of transformer, discussed next).

### Transformers

The transformer model architecture has been proven to be very suitable for fine tuning to tackle language tasks, with previous research [45] demonstrating that these models outperform other neural networks and standard classifiers. The RoBERTa-large-PM-M3-Voc model was chosen because of its proven capacity to understand clinical texts. Our initial training used 9 slightly imbalanced data sets, all with the same 932 positive labels but each with different 1127 negative labels, which, at 2059 records, was scarcely enough to fine-tune a transformer. However, we were able to establish which of the data sets worked best with the model, and this data set then became the foundation of data set development, which was chiefly undertaken to improve the performance of the transformers while providing a comparison to their performance improvements against other models.

The initial transformer model did no better than the pattern matching; its *F*_1_-score was 0.792, which was slightly less than the 0.797 of the pattern matching and much less than the 0.866 of the CNN-BiGRU model. Encouragingly, it matched the pattern matching model’s recall of 0.933, but it was let down by a relatively poor precision of 0.658. We found that this was mainly due to false positives; it was classifying many seizure-related records as febrile convulsion when they had no mention of fever. We added 112 more examples of negatively labeled seizure records, which we had manually checked to ensure fever was absent. The expectation was that the model could learn that seizure alone was not predictive of a febrile convulsion. However, this had only a slight effect; the *F*_1_-score only increased by 0.6 percentage points to 0.797, now equaling the pattern matching.

Therefore, we decided instead to add a lot more positive and negative records, as the model was clearly struggling with a lack of data to learn from. As we had no more positive records, we used a GPT-2 language model to generate synthetic examples of positive labels, as described previously [43]. Adding these enabled us to also sample and add many more negative labels to get a reasonably balanced data set of 5455 records, containing 2514 positive and 2941 negative labels—the slight imbalance was to give the model more negative examples. Training the transformer and other models just on the combined initial and synthetic data allowed us to measure the effect of the synthetic records clearly. Any extra negative records were randomly selected and not taken from the manually crafted 112 seizure negative records, which we had set aside at this point. This had a very positive effect; the *F*_1_-score on a newly fine-tuned model increased by 11.7 percentage points to 0.908, beating the 0.893 of the BiGRU, which was the best performing standard neural network trained on this version of the training data set.

Finally, we readded the 112 negative seizure records and reran our training for all models; the best transformer model now achieved an *F*_1_-score of 0.921, a more significant 1.6 percentage points improvement compared to the 0.6 increase obtained when the negative seizure records alone had been added in our previous experiment with them.

The best performing transformer model still maintained an impressive recall of 0.972, compared to its initial 0.993, but its precision had risen to 0.875 from 0.665. Its *F*_1_-score of 0.921 was 12.4 percentage points better than the pattern matching baseline of 0.797 and 2 percentage points better than the best standard neural network, which was the BiGRU with a score of 0.900.

This result confirmed our previous experience that transformers need more data to learn from compared to lighter-weight neural networks, but in the right conditions will outperform those simpler architectures. While the neural networks improved over their initial scores by around 4 percentage points following addition of synthetic data, the transformer improved from its starting score by >3 times that amount, at around 13 percentage points. The final transformer was superior to the final BiGRU by 2 percentage points (0.92 vs 0.90).

### Deployment

The best performing models from both standard neural networks and transformers were deployed in a Databricks environment. The transformer model was originally 1.4 GB in size and ran in a timely manner best on a graphics processing unit (GPU); inference was 5 times faster on a GPU versus a central processing unit (CPU). For performing inference on a CPU, we wanted to reduce both the memory required and the speed to perform inference. Therefore, we converted the best transformer checkpoint to an Open Neural Network Exchange (ONNX) model and optimized and then quantized it. Although the optimized model was no smaller than the original transformer model, it ran twice as fast as the transformer on a CPU. The quantized model was considerably smaller at 500 MB, had no loss of accuracy, and ran 40% faster again than the optimized model on a CPU; hence, it was used. Inference times on CPU for the quantized ONNX model were similar to the transformer model on GPU. However, inference times on CPU using the CNN-BiGRU, which was only 14 MB in size, were 10 to 12 times faster than the transformer on GPU and the quantized ONNX models.

The minimum available Databricks configuration was a Standard DS3 v2 CPU compute with 14 GB of memory and 4 cores and a 13.1 ML runtime, which costed 0.75 Databricks units per hour. All models were able to run on this. However, the much faster inference and smaller memory requirement of the standard neural network models meant that this configuration would be able to support the parallel loading of many such models (for the surveillance of numbers of syndromes), while based on the use of 2 transformer-based ONNX models, we estimate that it would support only up to 6 simultaneously loaded models before requiring a parallel deployment of computing capacity or a more powerful single capacity.

After 5 months of deployment, the model has predicted 749 febrile convulsion cases in the target group of children aged <6 years and 75,543 cases of no febrile convulsion in that group. To evaluate its performance, we sampled 125 of its predictions for each cohort. To ensure we had good candidates for potential false negatives, when sampling for the 125 nonfebrile convulsions, we filtered to records that had a mention of either febrile, seizure, or convulsion. Labeling resulted in 122 febrile convulsion and 128 nonfebrile convulsion records. The model had predicted incorrectly for 9 (3.6%) of the 250 records, resulting in a precision score of 0.952, a recall score of 0.975, and an *F*_1_-score of 0.964.

## Discussion

### Overview

The key objective of this study was to contribute to improving near real-time syndromic surveillance of febrile convulsions by using NLP models. We compared NLP approaches with a pattern matching baseline solution. We found that even with minimal initial training data but careful attention to the training examples and the addition of augmented data to improve the data, a transformer-based model could achieve superior performance, without needing any demanding text preprocessing or feature construction. We concluded that while the process of determining the best training data set was nontrivial, the result justified the effort and acted as a guide to further development for these models for classifying ED notes.

### Principal Findings

The format, quality, and length of ED triage notes can differ greatly, which presents a considerable challenge when it comes to text processing. To overcome these dissimilarities, one solution is to use lexicons to replace variations in spelling, abbreviations, and medical terms with standardized synonyms, and another solution is to use rules to recognize specific text features. Nonetheless, both these methods demand ongoing efforts to handle novel words or establish new rules, which can make the system more intricate as additional rules often need to be introduced to amend the impact of previously applied rules. In addition, the use of these methods can negatively affect the generalizability of solutions across hospitals, as terminology and abbreviations can be specific to individual ED departments.

In our research, we have demonstrated that neural networks and especially cutting-edge large language models can remove the need for preprocessing of text and can use text as is to achieve outstanding performance in syndromic surveillance of ED notes. Large language models have been originally trained on substantial volumes of text and have extensively learned complex textual patterns and relationships within texts, and when fine-tuned, they can quickly learn the specifics of previously unseen texts such as triage notes. This learning is enhanced if the model has been pretrained on similar texts, as was the case with the RoBERTa-large-PM-M3-Voc model we used, which had been trained on biomedical and clinical texts.

Development of supervised algorithms requires labeled data, which is hard to acquire [40,46]. Various techniques have been used by researchers to overcome this barrier. Researchers have employed various techniques to overcome this barrier, ranging from using proximal ICD codes [31], which suffers from a loss of expert targets, to the labor-intensive process of manually labeling many records [39]. Our strategy showed that use of language models for generating synthetic text is a highly effective and efficient way to augment data to improve the performance of the classification task. However, our findings suggested that although data augmentation can have a significant impact on the performance of language model–based classifiers, its impact on more conventional classifiers such as CNNs may be more limited. Specifically, augmenting data can substantially improve the accuracy and robustness of language model–based classifiers by expanding the data set and introducing greater variation in the data, particularly when there is information in the texts that clarifies the features of classes. However, there are lesser gains realized by standard neural networks and traditional classifiers, which indicates the greater ability of language models to benefit from textual clues.

Our findings also suggested that there is no single solution that can be universally applied for syndromic surveillance of ED triage notes, and simple pattern matching may provide reasonable performance, particularly where a syndrome can be clearly identified with the presence of specific keywords.

Clinical NLP research has been ongoing for several decades and has contributed significantly to many areas of patient care. However, despite these advances, there is still a lack of NLP systems that have been deployed and integrated into operational settings [47]. Our solution is currently deployed in a cloud-based environment and is continuously sending a stream of flagged presentations to an organization tracking adverse events following immunization monitor for possible increases against their background rate for detection of any vaccine safety signal related to febrile convulsions.

### Limitations

Our approach is extendible to similar scenarios; however, the model we created is specific to the task of detection of febrile convulsions in ED notes. Although the process of careful analysis, leading to an informed application of methods to enhance training data, is repeatable for the detection of other syndromes and potentially beyond (eg, vaccine adverse events following immunization), the approach depended on personal judgment and experience and was somewhat complex. More methodical approaches to determining optimal training data are described in the active learning literature [48], and our future focus will be on implementing these approaches while leveraging the insights gained from this study on using augmentation to enhance training data.

### Conclusions

Near real-time surveillance of febrile convulsion presentations to EDs is feasible using NLP solutions. We established that a large language model classifier can be trained in the context of few training examples by adding synthetically generated data and implemented into a real syndromic surveillance system, enabling surveillance of febrile convulsion following vaccination.

## Abbreviations

AEFI: adverse event following immunization
BERT: Bidirectional Encoder Representations from Transformers
BiGRU: bidirectional gated recurrent unit
CNN: convolutional neural network
CPU: central processing unit
ED: emergency department
GPU: graphics processing unit
GRU: gated recurrent unit
IDF: inverse document frequency
LSTM: long short-term memory
ML: machine learning
NLP: natural language processing
ONNX: Open Neural Network Exchange
SGD: stochastic gradient descent
SQL: structured query language
SVC: support vector classification
XG Boost: extreme gradient boosting
